# Microbead-based spoligotyping of *Mycobacterium tuberculosis* from Ziehl-Neelsen-stained microscopy preparations in Ethiopia

**DOI:** 10.1038/s41598-018-22071-9

**Published:** 2018-03-05

**Authors:** Barbara Molina-Moya, Mulualem Agonafir, Silvia Blanco, Russell Dacombe, Michel K. Gomgnimbou, Lizania Spinasse, Meissiner Gomes-Fernandes, Daniel G. Datiko, Thomas Edwards, Luis E. Cuevas, Jose Dominguez, Christophe Sola

**Affiliations:** 1 Hospital Universitari Germans Trias i Pujol, Institut d’Investigació Germans Trias i Pujol, Universitat Autònoma de Barcelona, Badalona, Spain; 2 CIBER Enfermedades Respiratorias (CIBERES), Instituto de Salud Carlos III, Madrid, Spain; 3 Ethiopian Health and Nutrition Research Institute, Addis Ababa, Ethiopia; 4 LSTM, Reach Ethiopia, Addis Ababa, Ethiopia; 5 College of Natural Sciences, Addis Ababa University, Addis Ababa, Ethiopia; 6 Liverpool School of Tropical Medicine, Liverpool, UK; 7 Institut de Biologie Intégrative de la Cellule (I2BC), CEA, CNRS, Univ. Paris-Sud, Université Paris-Saclay. Gif-sur-Yvette, Orsay, France; 8 Centre Muraz, Bobo-Dioulasso, Burkina Faso; 9 CAPES Foundation, Ministry of Education of Brazil, Brasília, Brazil; 10 REACH ETHIOPIA, Box 303 Hawassa, Ethiopia

## Abstract

The worldwide dissemination of *Mycobacterium tuberculosis* strains has led to the study of their genetic diversity. One of the most used genotyping methods is spoligotyping, based on the detection of spacers in the clustered regularly interspaced short palindromic repeats (CRISPR) locus. This study assessed the performance of a microbead-based spoligotyping assay using samples extracted from Ziehl-Neelsen-stained smear-microscopy preparations and described the genetic diversity of *Mycobacterium tuberculosis* among new TB patients in Southern Nations, Nationalities and Peoples’ Region (SNNPR) in Ethiopia. Among the 91 samples analysed, 59 (64.8%) generated spoligotyping patterns. Fifty (84.7%) samples were classified into 12 clusters (mostly Lineage 4 or 3) comprising 2–11 samples and nine had unique spoligotyping patterns. Among the 59 spoligotyping patterns, 25 belonged to the T1 sublineage, 11 to the T3-ETH, 5 to the URAL, 4 to the H3 and 14 to other L4 sublineages. There was a remarkable variation in genetic distribution in SNNPR compared to other regions of the country. Microbead-based spoligotyping is an easy-to-perform, high-throughput assay that can generate genotyping information using material obtained from smear microscopy preparations. The method provides an opportunity to obtain data of the *M. tuberculosis* genetic epidemiology in settings with limited laboratory resources.

## Introduction

Tuberculosis (TB) remains one of the most threatening infectious diseases, causing 10.4 million incident cases and 1.4 million deaths per year^1^. Ethiopia has the second largest population in Africa and has a high TB burden with an estimated incidence of 192 cases per 100,000 population^1^. The worldwide dissemination of *Mycobacterium tuberculosis* strains has led to the study of their genetic diversity, as lineages have differences in virulence, transmissibility, clinical presentation, capacity of acquiring drug resistance conferring mutations and possibly in the outcome of the disease^2,3^. The study of the genetic diversity of infections is also a valuable tool for local molecular epidemiology, contact tracing and outbreak management to identify recent transmission chains and prevent further spread of infections^3^. Several molecular methods are available to study *M. tuberculosis* genetic distribution. One of these, spoligotyping, is based on the PCR amplification of the genome’s clustered regularly interspaced short palindromic repeats (CRISPR) locus and the detection of spacers between the repeats by reverse hybridization. Reverse hybridization is usually performed on a nylon membrane using DNA extracted from culture or direct sputum and Zhang *et al*. adapted the process to microbeads^4^, which increases its throughput and makes it faster, easier to perform and cheaper. Although spoligotyping is popular in well-established laboratories, culture is not routinely performed in newly diagnosed patients of low-income settings and transport delays compromise the integrity of sputum for direct extraction. Few studies have reported it is possible to genotype *M. tuberculosis* from DNA recovered from Ziehl-Neelsen (ZN) stained microscopy preparations^5^. This approach would enable extending genotyping studies to a larger, less selected patient population, as most patients attend diagnostic laboratories only equipped with light microscopy. We therefore performed microbead-based spoligotyping directly from ZN-stained microscopy preparations to assess the proportion of samples that could be genotyped when specimens are collected under programmatic conditions in a remote population of Ethiopia, and analysed the genetic distribution of *M. tuberculosis* among new TB cases.

## Results and Discussion

**Figure 1:**
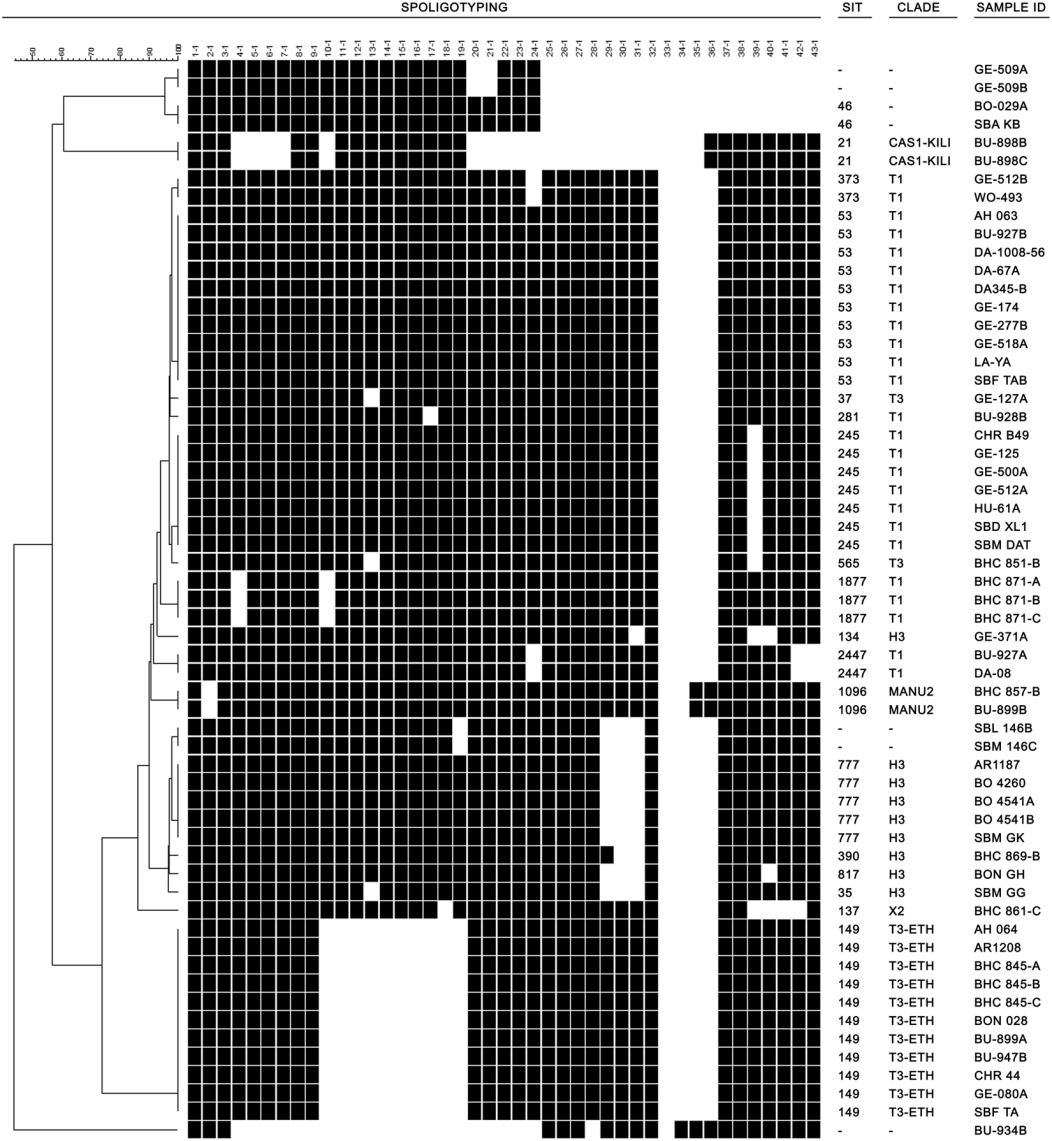
Bionumerics phylogenetic tree built on 59 spoligotypes obtained from positive Ziehl-Neelsen slides in Ethiopia, with spoligotype in binary format, spoligo-international type tags (SIT, according to SITVITWEB), sublineage and lineage designation and sample identification.

**Figure 2:**
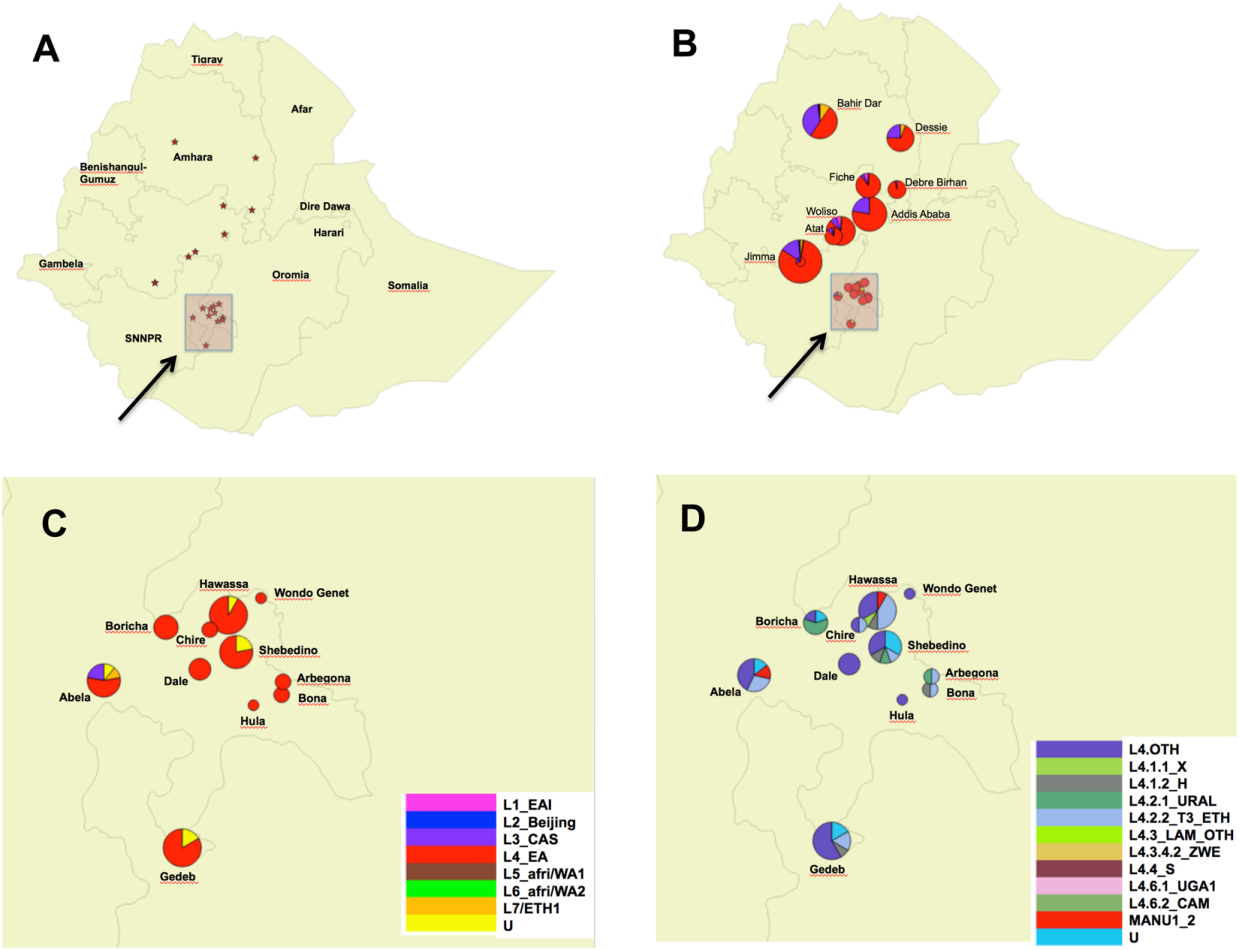
QGIS-built (v.2.18 Las Palmas, www.qgis.org) geographical genetical maps of Ethiopia. (**A**) map of Ethiopia with stars representing localities where data were produced (SNNPR) or collected from previously published papers^11,20–26^. (Supplementary file); (**B**) *Mycobacterium tuberculosis* complex lineage-based distribution map of Ethiopia. The rectangle show the precise investigation areas inside the Southern Nations, Nationalities and Peoples’ Region (SNNPR); (**C**) close-up of Map B; (**D**) close-up of *Mycobacterium tuberculosis* complex sublineages map distribution.

A total of 91 consecutive smear 2+ or 3+ samples were selected. Interpretable spoligotyping patterns (see Material and Methods) were obtained for 59 (64.8%) of the 91 DNA samples extracted. Of these, 50 (84.7%) were grouped in 12 clusters, each comprising between 2 and 11 samples and nine had unique patterns (Table 1, Fig. 1), resulting in 21 distinct patterns. Fifty-four samples (91.5%) were classified into seven previously described sublineages and five had three patterns for which a SIT had not been assigned by SITVITWEB^6^. The most common SITs were SIT149 (18.6%), SIT53 (16.9%) and SIT245 (11.9%), all belonging to lineage 4 (see close up Fig. 2C and D for the lineages and sublineages spatial distribution). The most common lineage was Euro-American (lineage 4), accounting for 85% of the samples. Lineage 3 accounted for 3.4% (n = 2) and lineage 7 for 1.7% (n = 1). Lineage 7 is represented by a spoligotype pattern with missing spacers 4–24 and was localized between ancient lineage 1 and modern lineages 2, 3 and 4 of *M. tuberculosis* phylogeny using genome sequencing^7,8^.

**Table 1:** Spoligotyping results of the 59 samples with interpretable results.

Lineage, sublineage	SIT (No. samples, % of the total samples)
Lineage 4, T1 (n = 25)	SIT 53 (n = 10, 16.9%)
	SIT 245 (n = 7, 11.9%)
	SIT 1877 (n = 3, 5.1%)
	SIT 373 (n = 2, 3.4%)
	SIT 2447 (n = 2, 3.4%)
	SIT 281 (n = 1, 1.7%)
Lineage 4, T3-ETH (n = 11)	SIT 149 (n = 11, 18.6%)
Lineage 4, URAL (n = 5)	SIT 777 (n = 5, 8.5%)
Lineage 4, H3 (n = 4)	SIT 35 (n = 1, 1.7%)
	SIT 134 (n = 1, 1.7%)
	SIT 390 (n = 1, 1.7%)
	SIT 817 (n = 1, 1.7%)
Lineage 3, CAS1-Kili (n = 2)	SIT 21 (n = 2, 3.4%)
Lineage unknown, MANU2 (n = 2)	SIT 1096 (n = 2, 3.4%)
Lineage 4, T3 (n = 2)	SIT 37 (n = 1, 1.7%)
	SIT 565 (n = 1, 1.7%)
Lineage 4, X2 (n = 1)	SIT 137 (n = 1, 1.7%)
Not assigned (n = 7)	SIT 46 (n = 2, 3.4%)
	Not described pattern (n = 2, 3.4%)
	Not described pattern (n = 2, 3.4%)
	Not described pattern (n = 1, 1.7%)

SIT: Spoligotyping International Type.

Among the total 59 spoligotyping patterns and considering the district of sample collection, 11 clusters that may be involved in TB transmission were detected: three were detected in Gedeb district (SIT245, T1, lineage 4, n = 3; SIT53, T1, lineage 4, n = 3; and SIT not assigned, lineage Unknown, n = 2); two in Hawassa district (SIT149, T3-ETH, lineage 4, n = 4; and SIT1877, T1, lineage 4, n = 3); two in Abela district (SIT21, CAS1-Kili, lineage 3, n = 2; and SIT149, T3-ETH, lineage 4, n = 2); two in Shebedino district (SIT245, T1, lineage 4, n = 2; and SIT not assigned, lineage Unknown, n = 2); a single cluster in Dale district (SIT53, T1, lineage 4, n = 3) and a single cluster in Boricha district (SIT777, URAL, lineage 4, n = 3).

The genetic diversity of *M. tuberculosis* in the Southern Region of Ethiopia in this study is partially different to that reported in other studies, which were mostly performed in central and north western regions. In a study from Northwest Ethiopia (Amhara Region), the major lineages identified were Euro-American (lineage 4) (51%), Central Asian (lineage 3) (35%) and lineage 7 (10%, which was inappropriately designated as *M. africanum* in some studies) and the most common SITs were SIT289 (17%), SIT134 (7%) and SIT3411 (5%)^9^. Interestingly, Lineage 7 accounted for 16% and 10% of the strains in two studies from Amhara Region^10,11^. Thus lineage 4 was more prevalent, whereas lineages 3 and 7 were less prevalent in the Southern Region than in other studies. The variation in genetic distribution in different regions of the country highlights the need of performing further studies, especially in regions where this information is scarce, such as the South of Ethiopia.

Studies including strains from all over Ethiopia are limited. In a recent study including 91 strains from selected sites from all over the country, the most common lineage was Euro-American (lineage 4) (75%), followed by Central Asian (lineage 3) (19%) and East-African Indian Lineage (lineage 1) (6%)^12^. In our study, lineage 4 was more prevalent whereas lineages 3 and 1 were less prevalent. Despite these differences, the most common SITs were similar (SIT53, 17%; SIT149, 12%) and all belonged to lineage 4^12^. Some differences may have arisen from the current difficulty to classify and assign some ancient spoligotyping (SIT54, SIT523, i.e. Manu 1 and 2, to a definite lineage). Finally, another study of 954 *M. tuberculosis* complex strains from all Ethiopia showed that the most common lineage was Euro-American (lineage 4) (71%), which was spread all over the country^8,13^. In contrast, lineage 3 was most prevalent in the northern region (47%), as well as lineage 7 (13%), whereas lineage 1 was most prevalent in the southern region^8,13^.

DNA extracted from ZN-stained slides has been used previously for spoligotyping, resulting in identical patterns as those obtained with cultured *M. tuberculosis* strains^14,15^. However, in countries where *M. africanum* may be prevalent and due to *M. africanum* exigent growth requirements, selection bias may have been introduced in molecular epidemiology studies^16^. Previous studies have reported sensitivities of 98.2% (95% CI: 71.1-98.4) of spoligotyping directly from positive ZN-stained slides, in comparison to culture^5^. However, we found a lower sensitivity, similar to that found in another study in Nigeria (Molina-Moya *et al*., unpublished) where 549 of 929 samples gave results (sensitivity 60%). This could be due to poor preservation of the samples and suboptimal DNA quality, linked to numerous sample transfer steps before DNA extraction was completed, which is a limitation of this study.

In settings where smear examination is the only diagnostic tool available, molecular diagnostics based on samples recovered from smears could be useful to generate novel genetic epidemiology information. Moreover, the possibility to perform spoligotyping directly on GeneXpert leftovers has been recently shown^17^.

The participants for this study were diagnosed solely using smear microscopy and the genetic distribution of the strains is partially different to that reported in other studies. These differences may be due to the different geographical location and the different classifications used, but may also reflect the less selected nature of the participants and thus highlight the need to develop methods that do not rely on highly selected patients in referral centres to obtain a better representation of the most common circulating genotypes in specific settings. The high clustering of samples, even if it should be complemented by higher discriminatory methods such as variable number of tandem repeats (VNTR) typing, indicates that recent transmission may have played an important role in this study. Spoligotyping may serve as a first-line screening test, with further analysis to confirm transmission dynamics.

In conclusion, microbead-based spoligotyping using DNA extracted from ZN-stained smear preparations had an acceptable diagnostic performance. Specimens collected under operational conditions in remote laboratories gave a snapshot of the *M. tuberculosis* strains circulating in a predominantly rural setting in Ethiopia and varied from those reported in other studies. Microbead-based spoligotyping from microscopy preparations is an easy-to-perform and high-throughput assay that could be useful to provide genotyping information in locations without culture facilities and further studies are warranted.

## Methods

The study was undertaken in Hawassa, Sidama and Gedio zones of the Southern Nations, Nationalities and Peoples’ Region (SNNPR) of Ethiopia (Fig. 2). Adults with cough of more than two weeks duration attending TB diagnostic health facilities who had not received treatment for TB were screened using direct smear microscopy by the local diagnostic laboratories. Sputum slides were stained with ZN according to WHO guidelines^18^ and examined using light microscopes. Smears graded as 2+ or 3+ were selected for spoligotyping. Samples were gathered as part of the routine clinical work for the diagnosis of TB. Slides processed by the diagnostic laboratories are routinely selected for external quality assurance (EQA) activities to monitor the performance of smear microscopy services. Culture and drug susceptibility testing were not available in the centres during the study period.

Smear-positive samples received by the reference laboratory were not considered infectious, but were processed in the hood of the TB culture laboratory to avoid cross-contamination of the material extracted. The mineral oil used for microscopy was removed in the reference laboratory using xylene and 25 μl of filtered TRIS-EDTA was added to the slides. Smears were scraped off using surgical blades and the material obtained was stored in Eppendorf tubes. Tubes were sent to Hospital Universitari Germans Trias i Pujol (Badalona, Spain) by courier at room temperature, where 75 μl of chelex (Bio-Rad Laboratories, USA) suspension were added in the hood of the laboratory. Samples were mixed thoroughly, incubated for 30 minutes at 95 °C, sonicated for 5 minutes and centrifuged for 15 minutes at 14000 g at 4 °C. The supernatant was transferred to a fresh microcentrifuge tube in the hood of the laboratory. Tubes were sent to Institut de Biologie Intégrative de la Cellule (Orsay, France) for spoligotyping. PCR was performed in 25 µl containing 5X Flexi buffer (Promega, France), 25 mM MgCl_2_, 2 mM dNTPs, 20 µM each primer (DRa-biotinylated and DRb), 0.25 µl of Go Taq DNA polymerase (Promega, France) and 5 µl of DNA. PCR was prepared in the hood of the laboratory to avoid cross-contamination. Amplification conditions were: 96 °C for 3 min; 25 cycles of 96 °C for 1 min, 55 °C for 30 sec and 72 °C for 15 sec; and 72 °C for 5 min.

Microbead-based hybridization was performed as previously described^4,19^. Analysis was done with a Luminex 200 system (Luminex Corp, Austin, TX, USA) and XPONENT software for LX100/LX200 (version 3.1.871.0). Interpretation of raw data was jointly done by two experts as previously described^20^. Interpretable spoligotyping patterns are those for which some spacers show a fluorescence that is several times higher than the cut-off (indicating the clear presence of those spacers) and other spacers show a fluorescence several times lower than the cut-off (indicating the clear absence of those spacers). An uninterpretable spoligotype pattern is a spoligotype for which (1) all spacers show a fluorescence several times lower than the cut-off (indicating the absence of all spacers) or (2) some spacers show a fluorescence close to the cut-off (where it is not possible to ascertain if they are present or absent) and some spacers show a fluorescence lower than the cut-off and it is not possible to ascertain if these spacers are truly absent or false negatives). Individual spoligotyping patterns were compared with the International Spoligotyping Database (SITVITWEB) of Pasteur Institute of Guadeloupe (http://www.pasteur-guadeloupe.fr:8081/SITVIT_ONLINE/)^6^. Spoligotyping International Types (SIT) and sublineages were assigned according to signatures provided in SITVITWEB. Numerical data were combined in Microsoft Excel and uploaded to BioNumerics version 6.1 (Applied Maths, Saint Marteen-Latem, Belgium).

All experimental protocols were approved by the Liverpool School of Tropical Medicine Research Ethics Committee and the Southern Region’s Health Bureau Ethics Committee of Ethiopia. The methods were carried out in accordance with the relevant guidelines and regulations. Informed consent was obtained from all participants and/or their legal guardian/s.

### Data availability

All data generated or analysed during this study are included in the published article.
